# Attentional Resource Allocation in Visuotactile Processing Depends on the Task, But Optimal Visuotactile Integration Does Not Depend on Attentional Resources

**DOI:** 10.3389/fnint.2016.00013

**Published:** 2016-03-08

**Authors:** Basil Wahn, Peter König

**Affiliations:** ^1^Neurobiopsychology, Institute of Cognitive Science, Universität OsnabrückOsnabrück, Germany; ^2^Department of Neurophysiology and Pathophysiology, Center of Experimental Medicine, University Medical Center Hamburg-EppendorfHamburg, Germany

**Keywords:** attentional load, multisensory integration, visual modality, tactile modality, attentional resources, visual search, tactile display

## Abstract

Humans constantly process and integrate sensory input from multiple sensory modalities. However, the amount of input that can be processed is constrained by limited attentional resources. A matter of ongoing debate is whether attentional resources are shared across sensory modalities, and whether multisensory integration is dependent on attentional resources. Previous research suggested that the distribution of attentional resources across sensory modalities depends on the the type of tasks. Here, we tested a novel task combination in a dual task paradigm: Participants performed a self-terminated visual search task and a localization task in either separate sensory modalities (i.e., haptics and vision) or both within the visual modality. Tasks considerably interfered. However, participants performed the visual search task faster when the localization task was performed in the tactile modality in comparison to performing both tasks within the visual modality. This finding indicates that tasks performed in separate sensory modalities rely in part on distinct attentional resources. Nevertheless, participants integrated visuotactile information optimally in the localization task even when attentional resources were diverted to the visual search task. Overall, our findings suggest that visual search and tactile localization partly rely on distinct attentional resources, and that optimal visuotactile integration is not dependent on attentional resources.

## 1. Introduction

In daily life, humans face tasks that are effortful and resource demanding such as looking for a person in a crowd or focusing to the sound of a person's voice in a noisy environment. In such tasks humans constantly process and integrate sensory input from multiple sensory modalities. However, the amount of sensory input that can be processed is limited by attentional resources. Specifically, via a process called “attention” only the sensory input that is most relevant for the current situation is selected for further processing (James, 1890; Chun et al., 2011).

A matter of ongoing debate is whether attentional resources are shared or distinct for the sensory modalities. That is, whether a task performed in one sensory modality (e.g., vision) draws from the same attentional resources as a second task performed in another sensory modality (e.g., haptics). The evidence for shared or distinct pools of attentional resources for the sensory modalities is conflicting. While many studies have shown that there are distinct attentional resources for each sensory modality (Duncan et al., 1997; Potter et al., 1998; Soto-Faraco and Spence, 2002; Alais et al., 2006; Hein et al., 2006; Talsma et al., 2006; Van der Burg et al., 2007; Arrighi et al., 2011; Wahn et al., 2015); many others have shown evidence for shared attentional resources (Jolicoeur, 1999; Arnell and Larson, 2002; Soto-Faraco et al., 2002; Arnell and Jenkins, 2004; Wahn and König, 2015a,b). It has been suggested that the distribution of attentional resources across sensory modalities depends on additional factors such as the type of tasks (Bonnel and Prinzmetal, 1998; Potter et al., 1998; Chan and Newell, 2008; Arrighi et al., 2011; Wahn and König, 2015a,b). In particular, when performing a spatial task in the visual modality and a discrimination task in the auditory modality, evidence for distinct attentional resources for the visual and auditory modality have been found (Arrighi et al., 2011). Also, when performing two discrimination tasks in separate sensory modalities, distinct attentional resources have been found (Alais et al., 2006). However, when performing a spatial task in the visual modality and another spatial task in the auditory modality, shared attentional resources for the visual and auditory modality have been found (Wahn and König, 2015a). Moreover, shared attentional resources were also found when a visual spatial task and a tactile spatial task were simultaneously performed (Wahn and König, 2015b). Taken together, studies suggest that tasks involving spatial attention draw from a shared pool of attentional resources while tasks involving the discrimination of stimulus attributes draw from distinct pools of attentional resources. What is more, when performing a spatial task and a discrimination task in separate sensory modalities, attentional resources are drawn from distinct pools of resources as well.

So far, a clear distinction between discrimination tasks and spatial tasks has been made. However, some tasks are more complex and involve spatial components as well as discriminative components. Specifically, in a visual search task, humans need to search for a target among distractors in a cloud of stimuli. Given the search target does not directly pop out (see Thornton and Gilden, 2007 for a discussion about serial and parallel processes in visual search), humans need to iteratively select an object in the visual field (i.e., allocate their attentional resources to objects in the visual field)—a spatial component of the task (Ghorashi et al., 2010; Eimer, 2014). Once an object is selected, humans then need to discriminate whether the attended object is the target or a distractor—a discriminative part of the task (Ghorashi et al., 2010; Eimer, 2014). What is more, it has been argued that these two components rely on separate processes (Ghorashi et al., 2010; Eimer, 2014).

Thus far, it has only been once investigated how attentional resources required for a visual search task are shared with tasks that are carried out in another sensory modality. In particular, in a study by Alais et al. (2006), a visual search task was performed in combination with either a visual or auditory discrimination task (i.e., a contrast or pitch discrimination task—note that in the same study also two discrimination tasks were performed in separate sensory modalities for which results indicated distinct attentional resources). Alais et al. (2006) found distinct attentional resources when tasks were performed in separate sensory modalities (i.e., vision and audition) while attentional resources were shared when both tasks were performed in the visual modality. In their study, the interference between performing a discrimination task and a visual search task was assessed. However, it is not known to what degree a spatial task performed in either the tactile or auditory modality does interfere with a visual search task. Given previous results that have shown that two spatial tasks performed in separate sensory modalities draw from a common pool of attentional resources (Wahn and König, 2015a,b), performing a visual search task and a spatial task should interfere with each other due to the spatial demands in a visual search task. However, given that a visual search task also involves a discriminative component, there is reason to believe that a visual search task and a tactile or auditory spatial task should not interfere with each other (Alais et al., 2006). The goal of the present study is to investigate whether the attentional resources required by a visual search task and a tactile localization task are shared or distinct.

So far, we have addressed processing of sensory input in separate sensory modalities and how processing within these separate sensory modalities could rely on shared attentional resources or distinct attentional resources. However, information from separate sensory modalities is rarely processed in isolation. Via a process called “multisensory integration” information from separate sensory modalities is combined into a unitary percept (Meredith and Stein, 1983; Stein and Stanford, 2008). It has been shown that the integrated information from separate sensory modalities is more accurate and more reliable (Ernst and Banks, 2002; Ernst and Bülthoff, 2004; Ernst, 2006; Helbig and Ernst, 2008). For instance, when humans need to localize an object in the world, they are more accurate and more reliable to localize the object when they receive visual as well as auditory information about the objects' location. What is more, it has been shown that humans integrate information from separate sensory modalities in a statistically optimal fashion (Ernst and Banks, 2002; Ernst and Bülthoff, 2004; Ernst, 2006), meaning they optimally combine the available information from different sensory modalities. From a technical perspective, if humans integrate the information from separate sensory modalities optimally, performance can be well predicted using the maximum likelihood estimation (MLE) model (Ernst and Bülthoff, 2004; Ernst, 2006).

Classically, multisensory integration has been considered to be an automatic process that occurs in a pre-attentive processing stage (Bloom and Lazeron, 1988). An everyday example is the so-called “ventriloquist effect” in which the voice of a ventriloquist appears to originate from the moving lips of a puppet. Here, an auditory signal (i.e., the voice of the ventriloquist) is automatically integrated with a visual signal (i.e., the moving lips of a puppet). The view that multisensory integration is an automatic process has been challenged by several studies (Alsius et al., 2005, 2007, 2014; Talsma and Woldorff, 2005; Talsma et al., 2007; Mozolic et al., 2008; Van der Burg et al., 2008; Fernández et al., 2015). In particular, it has been shown that attentional load can affect multisensory integration (Alsius et al., 2005, 2007, 2014; Mozolic et al., 2008). On the other hand, several studies have shown that the integration process is not affected by attentional load (Santangelo and Spence, 2007; Zimmer and Macaluso, 2007; Helbig and Ernst, 2008; Macaluso, 2010; Gentile et al., 2013; Wahn and König, 2015a,b, for a review, see Spence, 2010). One of the factors that has been suggested to account for the discrepancy in results are the type of stimuli that were used (Fernández et al., 2015; Talsma, 2015; Wahn and König, 2015a). In particular, multisensory integration was affected by attentional load predominantly in studies that used linguistic stimuli (Alsius et al., 2005, 2007, 2014; Mozolic et al., 2008). When using non-linguistic stimuli, multisensory integration in a discrimination task (Helbig and Ernst, 2008) and also in a spatial task was not affected by attentional load (Wahn and König, 2015a,b). In recent studies, we tested whether multisensory integration in a localization task depends on spatial attentional resources that are used for a secondary spatial task (i.e., a multiple object tracking task). In these studies, no effect of attentional load on visuotactile (Wahn and König, 2015b) and audiovisual integration (Wahn and König, 2015a) was observed. However, it is possible that finding an effect of attentional load depends on the type of the secondary task. So far, it has only been investigated whether multisensory integration is affected by attentional load of tasks involving linguistic stimuli (e.g., Alsius et al., 2005, 2007, 2014; Mozolic et al., 2008), discriminative tasks (Helbig and Ernst, 2008) or spatial tasks (Wahn and König, 2015a,b). Yet, it has not been investigated whether multisensory integration in a visuotactile localization task is affected if attentional resources are withdrawn via a simultaneously performed visual search task (i.e., a task that has both a discriminative and a spatial component), which is the goal of the present study.

Taken together, we will investigate two research questions. First, we will investigate whether the attentional resources required by a visual search task and a tactile localization task are shared or distinct. Second, we will investigate whether diverting attentional resources to a visual search task does interfere with visuotactile integration of visual and tactile location cues in a localization task. To this end, we will use a dual task paradigm in which a localization task and a visual search task are performed separately or at the same time. In the localization task, participants receive in separate conditions visual, tactile, or redundant visual and tactile location cues that they are required to localize. The tactile location cues are received via a vibrotactile belt worn around the waist of participants as previous studies have shown that spatial information received via a vibrotactile belt can be intuitively interpreted and accurately localized (Wahn and König, 2015b; Wahn et al., 2015). The visual search task is a serial self-terminated visual search task in which participants need to search for a target among inhomogeneous distractors. Similar to the study by Alais et al. (2006), the visual search is confined to a small number of visual degrees in the center of the screen (i.e., two visual degrees), requiring attentional processing of visual information only in foveated regions in the center of the visual field and not in the visual periphery (but see Corbetta, 1998; de Haan et al., 2008 for commonalities between visual processing in the fovea and periphery). In the dual task condition, participants perform the visual search task in combination with one of the localization task conditions (i.e., the required attentional resources are increased). In particular, while searching, they need to simultaneously localize either visual, tactile, or redundant visual and tactile location cues. By varying the sensory modality in which the localization task was performed, we varied from which sensory modality attentional resources are additionally recruited. If attentional resources required for the visual search task and tactile localization task are shared, we expect that the interference between these tasks in the dual task condition is equal to the interference between tasks when the visual search task is performed together with the visual localization task. Conversely, if attentional resources are distinct, the interference should be lower. In particular, performing a visual search task in combination with a tactile localization task should yield a lower interference between tasks than performing the visual search task in combination with a visual localization task. With regard to the question whether diverting attentional resources to a visual search task does interfere with visuotactile integration, if visuotactile integration in the localization task is not disrupted by diverting attentional resources to the visual search task, then participants should integrate the redundant visual and tactile spatial cues in the localization task regardless of whether they simultaneously perform the visual search task or not. However, if visuotactile integration is affected by performing the visual search task, then localization cues should be no longer integrated. That is, localization performance when receiving redundant visual and tactile localization cues should not be more accurate than receiving the more reliable of the two localization cues.

## 2. Methods

### 2.1. Methods of data acquisition

#### 2.1.1. Participants

Twelve students (9 female, *M* = 26.42 years, *SD* = 9.05 years) of the University of the Osnabrück participated in this study. The study was approved by the ethics committee of the University of the Osnabrück. We informed participants about their rights and all participants signed a written consent form. Participants either received a monetary reward or subject hours for participation.

#### 2.1.2. Experimental setup

Participants sat in a dark room in front of a computer screen (BenQ XL2420T, resolution 1920 × 1080, 120 Hz, subtending a visual field of 32.87 × 18.49 visual degrees) at a distance of 90 cm. They wore a self-constructed vibrotactile belt around the waist with the vibromotors placed on the stomach (minimum of 4 cm between vibromotors). Vibromotors were precision microdrives (14 mm diameter, vibration frequency 170–185 Hz). Participants' eye movements were recorded using an Eyelink 1000 (monocular tracking, 250 Hz sampling rate). In order to calibrate eye position, we used a 14-point grid and repeated calibration until the mean error was below 0.7 degrees.

#### 2.1.3. Experimental conditions and experimental procedure

**Figure 1:**
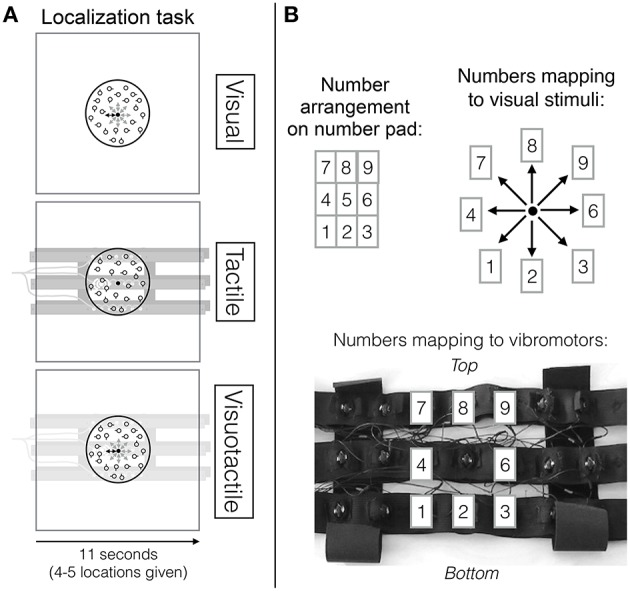
**(A)** Localization task overview. The top row depicts the visual localization task (in which visual location cues were received), the middle row the tactile localization task (in which tactile location cues were received) and the bottom row the visuotactile localization task (in which visual and tactile location cues that point to the same direction were received). **(B)** Mapping of number pad (top left) to visual spatial cues on the screen (top right) and to tactile spatial cues (bottom). This figure has been adapted from our previous study (Wahn and König, 2015b) with permission of Koninklijke Brill NV.

In the experiment, participants performed either a visual search task, a localization task or both tasks at the same time. In the visual localization task, the fixation point in the center of the screen changed position (distance between position changes was 0.04 visual degrees) to one out of eight possible positions surrounding the central position and then changed its position back to the central position. Participants' task was to indicate position changes using a corresponding key on the number pad immediately after a location cue was received (see top row in Figures 1A,B). Within a trial, 4–5 position changes were indicated and the onsets of these changes were randomly chosen with a minimum time of 1 s between onsets and position changes lasted 600 ms, requiring participants to continuously pay attention to the localization task. Given the high pace at which the location cues were received, a time limit for detecting a location cue was not set. Location cues were received continuously throughout a trial regardless of whether participants failed to respond to a cue. A trial in which only the localization task was performed always lasted 11 s. Analogously, in the tactile localization task, the central vibromotor of the vibrotactile belt vibrated continuously and then the vibrating vibromotor briefly changed to one of eight possible other vibromotors surrounding the central vibromotor (see middle row Figure 1A and bottom row Figure 1B). Position changes also lasted 600 ms. Again, these onsets were randomly chosen, leaving a minimum time of 1 s between onsets, again totalling 4–5 position changes per trial. When both types of location cues (i.e., the visual and the tactile locations cues) were presented simultaneously, the location change was always congruent and occurring at the same time.

**Figure 2:**
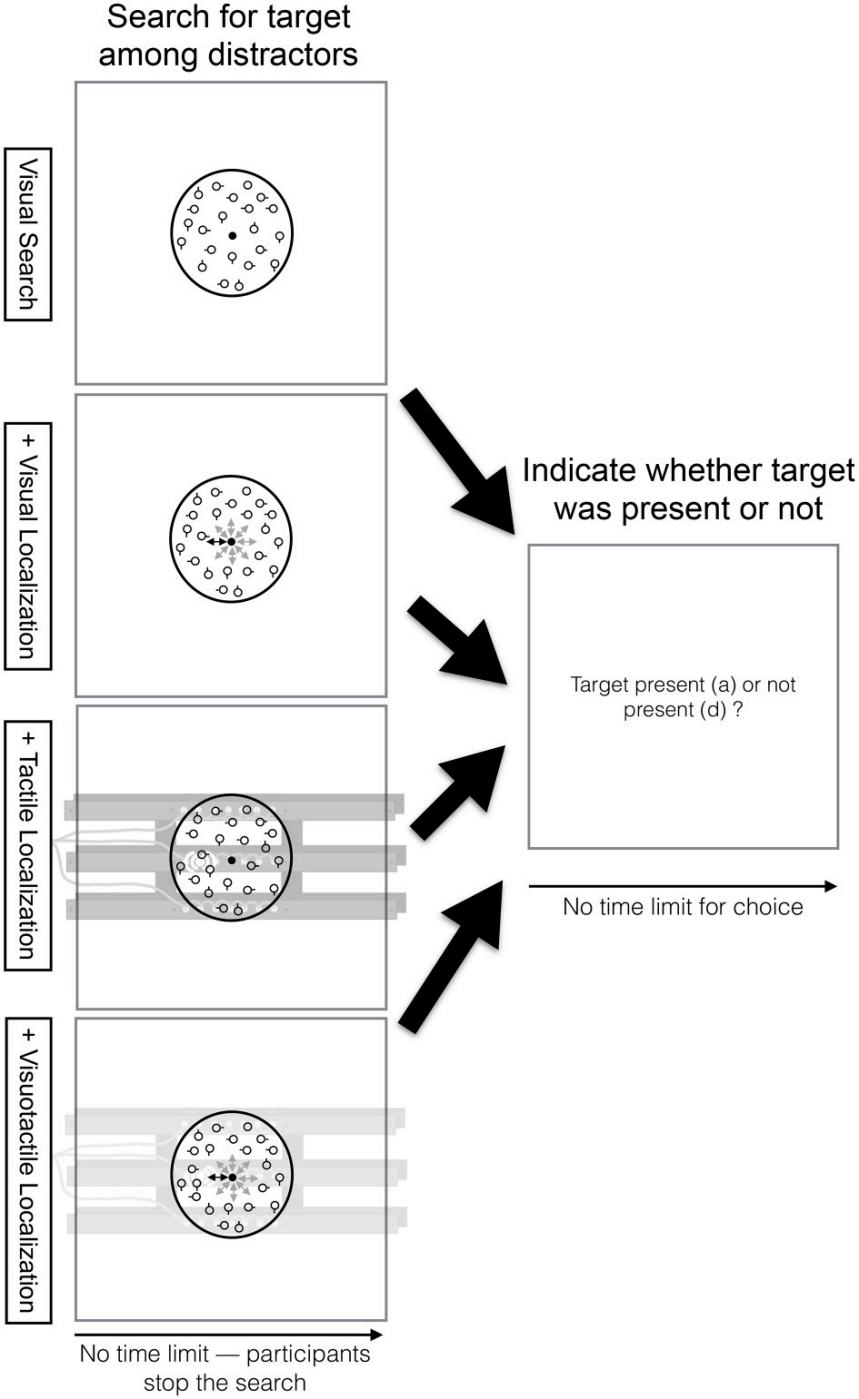
**Visual search task time course overview**. Participants searched within a circle (2 visual degree diameter) in the center of the screen for a target among distractors (see top row, first column). Within the circle, there were always 20 circles (0.13 visual degrees radius) shown of which one was a target in 50% of the trials. Distractors differed from the target in one feature. They had an additional short line (0.05 visual degrees) attached to it (either at 0°, 90°, 180°, or 270°). Participants could stop the search at any point in the trial by pressing the “s” key on the keyboard. Once participants pressed this key, they were required to indicate whether the target was present or not (see top row, second column). Participants did not receive feedback whether they answered this question correctly. The visual search task was performed in combination with the visual (2nd row), tactile (3rd row), or visuotactile localization task (4th row). Note that there was no limit for the location cues—location cues were received as long participants were searching. Yet, participants only performed the localization task while searching for the target among distractors. Once participants stop the search, they no longer need to do the localization task.

In the visual search task (see top row, Figure 2), participants searched within a circle (2 visual degree diameter) in the center of the screen for a target among distractors. Within the circle, there were always 20 circles (0.13 visual degrees radius) shown of which one was a target in 50% of the trials. Distractors differed from the target in one feature. They had an additional short line (0.05 visual degrees) attached to it (either at 0°, 90°, 180°, or 270°). Participants could stop the search at any point in the trial by pressing the “s” key on the keyboard. Once participants pressed this key, they were required to indicate whether the target was present or not. Participants did not receive feedback whether they answered this question correctly.

In the dual task conditions, participants performed the visual search task and the localization task concurrently. In particular, while participants were searching, they had to continuously localize the location cues in the localization task (i.e., there was no limit for the location cues—location cues were received as long participants were searching). Once participants stop the search, they no longer need to do the localization task. The visual search task was performed in combination with either the visual, tactile, or visuotactile localization task (see 2nd, 3rd, and 4th row of Figure 2, respectively).

Importantly, given that humans can only foveate two visual degrees at a time (Fairchild, 2013), the visual search task is carried out only in the central two visual degrees of the screen in order to avoid any confounds due to visual scanning. In particular, we want to avoid that participants repeatedly switch their gaze between the visual localization task and the visual search task only because they are too far apart in the visual field. Here, the visual localization task is performed in the center of the screen and is surrounded by the visual search task. When participants fixate at the center of the screen or next to it within the confines of the visual search task, both tasks are visible within the foveated two visual degrees in the visual field.

The experiment was divided into 21 blocks each consisting of 10 trials, presented in a pseudorandomized order. In one block, participants always performed the same condition, which was indicated at the beginning of each block. Each set of seven blocks included all seven conditions (i.e., the visual search task, visual localization task, tactile localization task, visuotactile localization task, and the visual search task combined with each type of localization task). Repetition of a condition in consecutive blocks was avoided. After every seventh block, we offered participants an optional break. The entire experiment took about 2 h.

Prior to the experiment, participants were doing four training trials for each task and task combination to become affiliated with the tasks. After training trials in the tactile and visual localization task were performed, performance was checked whether it reached ceiling or floor performance and participants were asked whether they felt that the two tasks were unequal in difficulty. When at least one of these was the case, we adjusted the task difficulty of the visual localization task by increasing or decreasing the distance between the central location cue and the location cues surrounding the central location cue. Overall, we decreased the distance between location cues (from 0.04 to 0.02 visual degrees) for two participants as they almost reached a ceiling performance and also felt that the visual localization task was easier than the tactile localization task.

### 2.2. Methods of data analysis

Data was minimally pre-processed. With regard to the search times, we used the median as a robust estimator of the central tendency as search times tend to be heavily influenced by outliers. In particular, we took the median across search times for each participant for each condition. With regard to the other dependent variables, we used the mean as an estimator of central tendency. With regard to the eye tracking data, we calculated the median gaze deviation from the center of the screen for each trial. Here, we again chose the median as a robust estimator of central tendency as it is not influenced by implausible data (e.g., as produced by eye blinks).

As statistical tests, repeated measures ANOVAs were computed followed by *post-hoc* paired *t*-tests. When the assumption of normality was violated (assessed with a Shapiro-Wilk-Test, alpha = 0.05), the respective dependent variable was transformed using the natural logarithm. When the sphericity assumption of the ANOVA was violated (assessed with Mauchly's Test for Sphericity, alpha = 0.05), a Greenhouse Geisser correction was applied. All graphics were generated using ggplot2 (Wickham, 2009). For *post-hoc* tests, in order to account for multiple comparisons, we used the Holm-Bonferroni method (Holm, 1979).

## 3. Results

### 3.1. Do visual search and tactile localization share attentional resources?

With regard to the visual search task, we first tested whether participants deviated their gaze more than one visual degree from the center of the screen when simultaneously performing the localization task. If participants would deviate their gaze from the center of the screen to such a large extent (as a consequence of doing the visual search task), then they could no longer foveate the visual localization task at the same time, which would require participants to alternate their gaze between the two tasks. As a consequence, participants could systematically take longer to do the visual search task in this particular condition only due to the need to alternate their gaze between tasks. For this purpose, we calculated the median gaze deviation (in visual degrees) from the center of the screen for each trial. We tested for each condition whether the gaze deviated more than one visual degree from the center of the screen with one sample *t*-tests. We found in all cases that participants deviated their gaze less than one visual degree from the center [visual search alone: *M* = 0.65°, *t*_(11)_ = −11.71, *corrected p* < 0.001, visual search in combination with visual localization: *M* = 0.57°, *t*_(11)_ = −8.76, *corrected p* < 0.001; visual search in combination with tactile localization *M* = 0.65°, *t*_(11)_ = −7.13, *corrected p* < 0.001; visual search in combination with visuotactile localization: *M* = 0.62°, *t*_(11)_ = −7.07, *corrected p* < 0.001], indicating that the visual search task did not require participants to deviate more than one visual degree from the center of the screen. What is more, it could be that due to visual attentional resource limitations participants deviated their gaze less from the center when performing the visual search task in combination with the visual localization task than when they performed it in combination with the tactile or visuotactile localization task. We tested whether this is the case by conducting a one way repeated ANOVA with the factor condition and the dependent variable visual degrees. We found a significant main effect [*F*_(1.51, 16.66)_ = 5.60, *p* = 0.016]. *Post-hoc* tests yielded that participants deviated their gaze less from the center when they performed the visual search task in combination with the visual localization task than when they performed it alone [mean difference: 0.08°, *t*_(11)_ = 3.19, *corrected p* = 0.034], in combination with the tactile localization task [mean difference: 0.07°, *t*_(11)_ = 6.65, *corrected p* < 0.001] or visuotactile localization task [mean difference: 0.05 °, *t*_(11)_ = 4.22, *corrected p* = 0.007]. These results indicate that participants could not simultaneously attend the visual localization task and at the visual search task [other comparisons were not significant (*corrected ps* > 0.363)].

**Figure 3:**
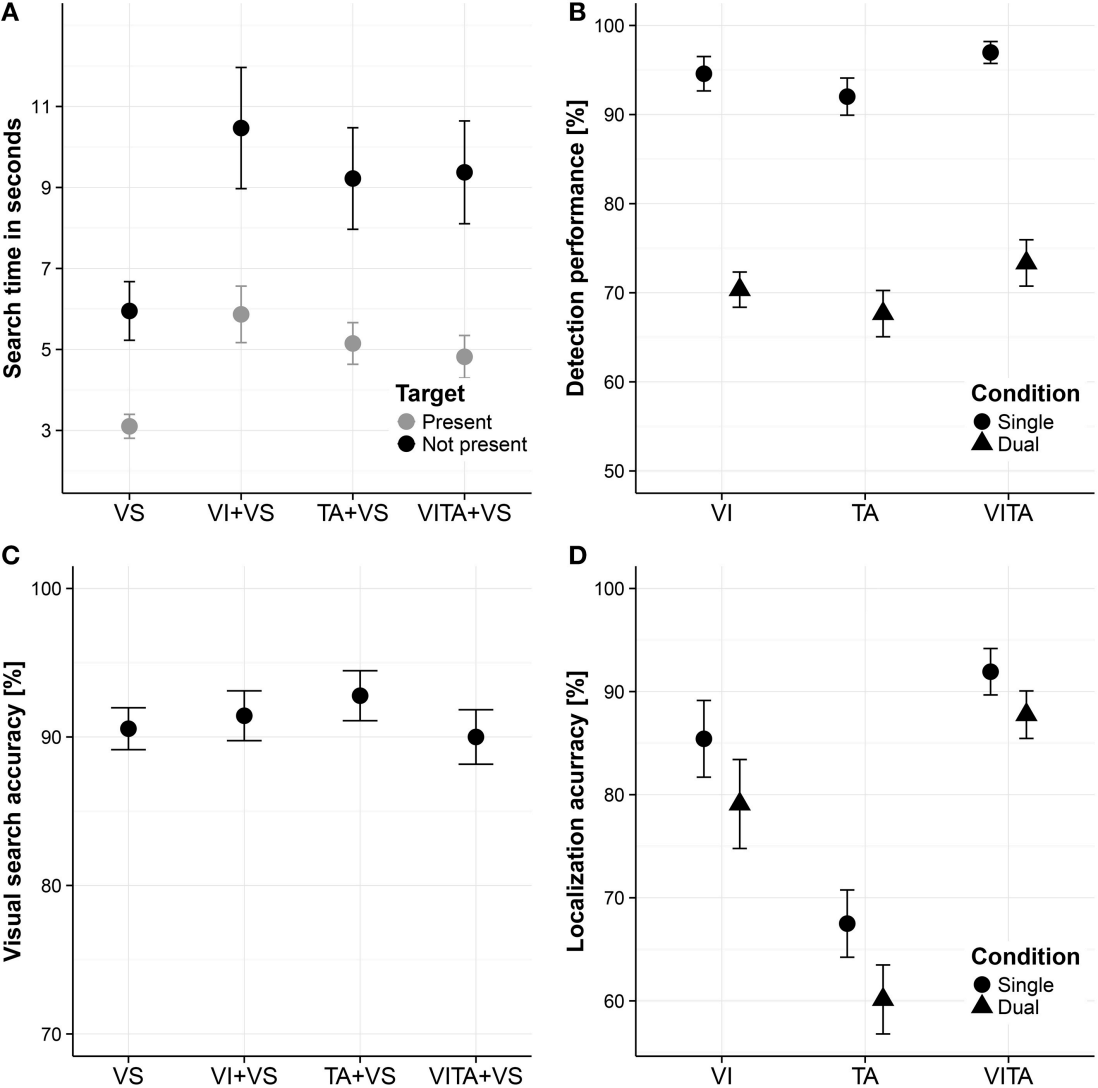
**Visual search task and localization task results**. **(A)** Visual search times for visual search task performed alone (“VS”), performed in combination with a visual localization task (“VI+VS”), tactile localization task (“VI+TA”) or visuotactile localization task (“VITA+VS”). **(B)** Detection performance for localization task (i.e., fraction of spatial cues that are detected without necessarily localizing them correctly) for the visual (“VI”), tactile (“TA”) and visuotactile (“VITA”) localization task for the single and dual task condition (i.e., when the visual search task is simultaneously performed), referred to as “single” and “dual,” respectively. **(C)** Visual search accuracy (i.e., whether a target is present or not) for VS, VI+VS, TA+VS, and VITA+VS. **(D)** Localization accuracy for detected location cues (i.e., fraction correct of the detected spatial cues) for VI, TA, and VITA for the single and dual task condition, referred to as “single” and “dual,” respectively. Error bars in all panels are standard error of the mean.

In order to assess whether attentional resources are shared between the visual and tactile modality, we compared median search times between the dual task conditions (for a descriptive overview, see Figure 3A). To obtain a measure of how much the localization task interfered with the search task, we subtracted the search times when the visual search task was performed alone from the search times in the dual task conditions. The calculated differences were all well above zero, indicating that the localization task considerably interfered with the search task [*M* = 2.96 s, *t*_(11)_ = 6.48, *p* < 0.001]. In order to test whether the interference depended on the modality in which the localization task was carried out, we analyzed the data using a two factorial repeated measures ANOVA with the factors target (target present or target not present) and modality (visual, tactile or visuotactile) with the dependent variable search time. We found a significant main effect of target [*F*_(1, 11)_ = 8.25, *p* = 0.015], indicating that participants stopped the search earlier when a target was present than when it was not present. Moreover, we found a significant main effect of modality [*F*_(2, 22)_ = 6.49, *p* = 0.006], suggesting that search times in the dual task conditions depended on the modality in which the localization task was carried out. We did not find a significant interaction between the two factors [*F*_(2, 22)_ = 0.89, *p* = 0.424], which indicates that the differences between the levels of the factor modality do not depend on the factor target. In particular, differences between the modality conditions are not significantly altered by whether a target was present in the search task or not. For the purpose of *post-hoc* tests, we averaged search times across the levels of the factor target for each type of modality condition and performed pairwise comparisons between all levels of the factor modality. We did not find a significant difference when comparing the tactile and visuotactile condition [mean difference: 0.08 s, *t*_(11)_ = 0.30, *corrected p* = 0.773]. For the critical comparisons, we found a significant difference between the visual and tactile [mean difference: 0.98 s, *t*_(11)_ = −2.61, *corrected p* = 0.048], and visual and visuotactile condition [mean difference: 1.07 s, *t*_(11)_ = −3.45, *corrected p* = 0.016]. Participants searched about 1 s faster when simultaneously performing a tactile localization task or visuotactile localization task in comparison to simultaneously performing a visual localization task. In comparison to the overall interference between tasks of about 3 s, performing the search task and localization task in separate sensory modalities, reduced the interference between tasks by about 33%.

However, a possible confound could be that participants systematically traded-off search speed for search accuracy (i.e., a shift in the decision criteria when to end the search) in one of the dual task conditions. Specifically, when simultaneously performing the tactile or visuotactile localization task and the visual search task, participants could choose to search not as thoroughly in these conditions as when simultaneously performing the visual localization task and the visual search task. As a consequence, participants could systematically stop their search earlier, resulting in faster search times for these conditions. In order to assess this possibility, we computed the visual search accuracy (i.e., fraction correct of whether searches were correctly classified as target present or not present) in the visual search task and compared these values for the single task condition and all dual task conditions. On a descriptive level, search accuracies did not greatly differ between conditions (see Figure 3C). Using search accuracy as the dependent variable, we performed a one factorial repeated measures ANOVA with the factor condition including the single task condition (performing the visual search alone) and all dual task conditions (performing the visual search either in combination with the visual, tactile or visuotactile localization task). We did not a find a significant main effect of condition [*F*_(3, 33)_ = 0.94, *p* = 0.431], which suggests that participants did not systematically traded-off search speed for search accuracy in any of the conditions. On a descriptive level, there is even a slight indication that participants searched more accurately when performing the visual search task together with the tactile localization task than with the visual localization task.

Moreover, for the question whether there are shared or distinct attentional resources for the visual and tactile modality, it does not suffice to only evaluate performance in the visual search task. Performance in the localization task needs to be evaluated as well in order to rule out whether there is an asymmetric allocation of attentional resources across tasks. For instance, when simultaneously performing the tactile or visuotactile localization task with the search task, it could be that participants devoted considerably more attentional resources to the search task than to the localization task. Conversely, when participants were performing the visual localization task and the visual search task at the same time, they could have distributed their attentional resources more evenly across tasks. An uneven distribution of attentional resources across tasks could result in a search benefit or localization performance benefit for any of the dual task combinations that is independent of the sensory modality in which the localization task is performed. In order to assess this possibility, we analyzed the detection performance of spatial cues (i.e., fraction of spatial cues that are detected without necessarily localizing them correctly—“detection performance,” for a descriptive overview see Figure 3B) in the localization task and the fraction correct of the detected spatial cues (“localization accuracy,” for a descriptive overview, see Figure 3D). For the detection performance, a key press on the number pad that was recorded between the onsets of location cues was regarded as a hit.

With regard to the detection performance (Figure 3B), we performed a two factorial repeated measures ANOVA with the factors task (single, dual) and modality (visual, tactile, and visuotactile). We found a significant main effect of task [*F*_(1, 11)_ = 115.75, *p* < 0.001], indicating that the visual search task interfered with the ability to detect spatial cues in the localization task. Moreover, we found a significant main effect of modality [*F*_(2, 22)_ = 5.72, *p* = 0.001], suggesting that the ability to detect spatial cues differs between modality conditions. However, we did not find a significant interaction effect [*F*_(2, 22)_ = 0.04, *p* = 0.959], indicating that the effect of the dual task did not differ between modality conditions. This result suggests that participants did not distribute attentional resources unevenly across tasks. For the purpose of *post-hoc* tests, we averaged across the levels of the factor task for each modality condition and conducted pairwise comparisons between modality conditions. Comparisons yielded that participants detected significantly more spatial cues in the visuotactile condition in comparison to the tactile condition [*t*_(11)_ = 3.43, *corrected p* = 0.017], suggesting a multisensory benefit for receiving redundant spatial cues from the visual and tactile modality. The other comparisons were not significant [visual vs. tactile condition: *t*_(11)_ = 1.47, *corrected p* = 0.169; visual vs. visuotactile condition: *t*_(11)_ = 1.99, *corrected p* = 0.143].

With regard to the accuracy of the detected spatial cues (Figure 3D), we used the same two factorial repeated measures ANOVA design as for the dependent variable detection performance. Factors are again task (single, dual) and modality (visual, tactile, visuotactile). We found a significant main effect of task [*F*_(1, 11)_ = 21.03, *p* < 0.001], indicating that the visual search task interfered with the ability to localize the spatial cues. Moreover, we found a significant main effect of modality [*F*_(1.37, 15.04)_ = 28.52, *p* < 0.001], suggesting that participants' ability to localize the spatial cues differed between modalities. We did not find a significant interaction effect [*F*_(1.37, 15.08)_ = 0.36, *p* = 0.624], again indicating that the effect of the dual task did not differ between modality conditions. The absence of an interaction effect suggests that participants did not distribute attentional resources unevenly across tasks. For the purpose of *post-hoc* comparisons, we averaged across the levels of the factor task for each modality condition and performed pairwise comparisons between the modality conditions. We found that participants had a significantly higher performance in localizing the spatial cues in the visuotactile condition in comparison to the visual [*t*_(11)_ = 2.79, *corrected p* = 0.018] and the tactile condition [*t*_(11)_ = 8.55, *corrected p* < 0.001], suggesting that having redundant spatial cues from the visual and tactile modality leads to a better localization performance. Moreover, we found a significant difference between the visual and tactile condition [*t*_(11)_ = 4.01, *corrected p* = 0.004], suggesting that the tactile localization task was more difficult than the visual localization task.

In summary, participants performed the search task faster in combination with the tactile or visuotactile localization task in comparison to performing the visual search task in combination with the visual localization task. The superior search performance was not the result of allocating more attentional resources to the visual search task than to the localization task. In addition, these results cannot be explained by a shift of decision criteria in the search task that is specific to one of the modality conditions. Overall, findings indicate that performing a search task and localization task in separate sensory modalities (i.e., vision and haptics) leads to a better performance than performing the two tasks in the same sensory modality (i.e., vision). However, regardless of the sensory modalities in which task were performed, tasks also considerably interfered.

### 3.2. Is optimal visuotactile integration dependent on attentional resources?

In order to assess whether visuotactile integration is dependent on attentional resources, we tested whether the predictions of multisensory cue integration are fulfilled in the single task and dual task condition. The predictions of cue integration are that integrated cues lead to less variable location estimates than the unimodal location estimates (i.e., when either the visual or tactile location cues are received alone). Conversely, if the predictions of cue integration are not fulfilled, the variance for the location estimates when receiving redundant visual and tactile location cues should not be lower than the variance when only the better of the two location estimates is received (Ernst and Banks, 2002; Ernst and Bülthoff, 2004; Ernst, 2006).

**Figure 4:**
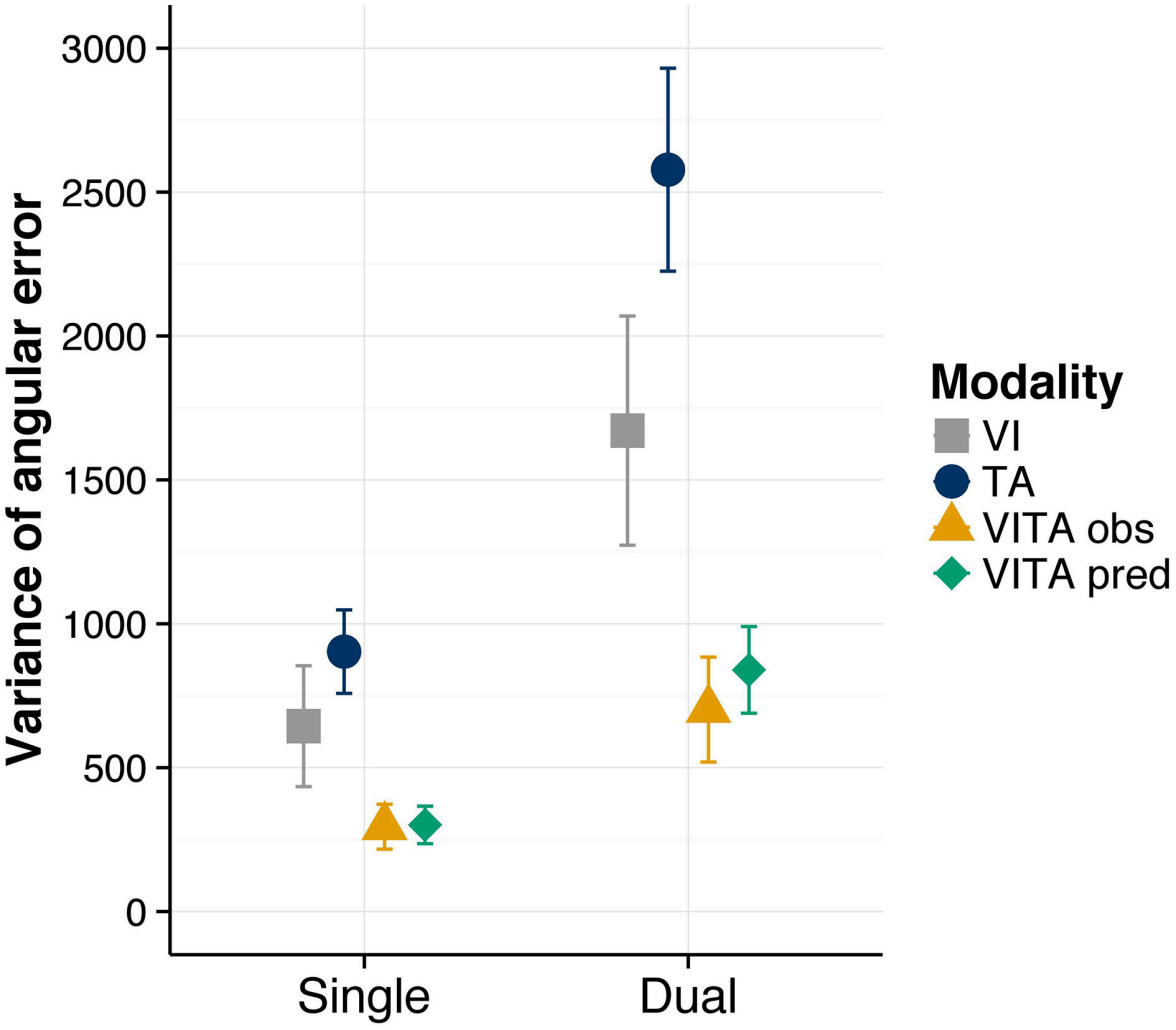
**Variance of angular error results**. Observed variance of angular error for the visual (“VI”), tactile (“TA”), and visuotactile (“VITA obs”) localization task. “VITA pred” refers to the variance predicted by the MLE model (Ernst and Banks, 2002). Localization task modality is shown separately for the single and dual task conditions. Error bars are standard error of the mean.

In order to have a measure of the variance of the location estimates in localization task, we first calculated for each trial the signed angular error between a given location cue and the chosen cue. For instance, if the indicated location cue would be the top direction (i.e., corresponding to the eight on the number pad) and participants would indicate the right direction (i.e., corresponding to the six on the number pad) they would commit an error of 90°. Conversely, if they would indicate the left direction (i.e., corresponding to the four on the number pad), they would commit an error of −90°. We then computed for each condition and participant the variance of the signed angular error as a dependent measure. On a descriptive level (see Figure 4), the variance is considerably reduced for the visuotactile localization task in comparison to the visual and tactile localization task. This reduction is also present for the dual task condition, suggesting that the dual task did not interfere with the integration process. We tested whether these observations were statistically reliable by again computing a two-way repeated measures ANOVA with the factors task (single, dual) and modality (visual, tactile, and visuotactile). We found a significant main effect of task [*F*_(1, 11)_ = 40.11, *p* < 0.001], indicating that the dual task increased the variance of the location estimates. Moreover, we found a significant main effect of modality [*F*_(1.81, 19.95)_ = 12.98, *p* < 0.001], suggesting that the variance of the location estimates differed between conditions. However, we did not find a significant interaction effect [*F*_(2, 22)_ = 2.92, *p* = 0.075]. For the purpose of *post-hoc* tests, we averaged across the levels of the factor task for each modality condition and performed pairwise comparisons between the modality conditions. We found significant comparisons between the visuotactile condition and the visual condition [*t*_(11)_ = 3.17, *corrected p* = 0.018] and the tactile condition [*t*_(11)_ = 5.12, *corrected p* = 0.001], indicating that the variance of location estimates was significantly reduced in the visuotactile condition [the comparison involving the visual and tactile condition showed a trend toward significance *t*_(11)_ = −2.11, *corrected p* = 0.059].

In sum, results indicate that receiving redundant location cues from the visual and tactile sensory modalities lead to a reduction in variance in comparison to receiving unimodal location cues. This reduction in variance is robust against additional attentional load in the dual task condition, indicating that visuotactile integration is not dependent on attentional resources required by a visual search task.

In addition to testing whether the variance of location estimates is reduced, we also tested whether the formal predictions of optimal cue integration fit our data. When cues from multiple sensory modalities are integrated in a statistically optimal fashion, the variance of estimates in a multisensory condition can be predicted by the variances of the estimates in the unisensory conditions using the following formula of the MLE model (Ernst and Banks, 2002; Ernst and Bülthoff, 2004; Ernst, 2006):

(1) $$\begin{array}{l} \sigma_{visuotactile}^{2}=\frac{\sigma_{visual}^{2}*\sigma_{tactile}^{2}}{\sigma_{visual}^{2}+\sigma_{tactile}^{2}} \end{array}$$

We applied this formula to our data, separately for the single and dual condition (i.e., estimates of the variances were taken from the unisensory localization tasks for the single and dual task conditions, respectively), and found that the predictions of optimal cue integration closely match the variance estimates in our data (see Figure 4). This finding suggests that participants integrated the cues optimally regardless of whether they had to perform the visual search task in addition to the localization task or not.

Finally, we also addressed whether these results could alternatively be explained by a speed accuracy trade-off. In particular, it could be that participants systematically took longer to respond to the visuotactile location cues than to the visual or tactile location cues and thereby gained an accuracy benefit. For this purpose, we analyzed the reaction times for correctly localized location cues with a two way repeated measures ANOVA with the factors task (single, dual) and modality (visual, tactile, and visuotactile). We found a significant main effect of task [*F*_(1, 11)_ = 19.80, *p* < 0.001], indicating that the task significantly slowed down reaction times in the dual task condition (mean difference: 0.05 s). Moreover, we found a significant main effect of modality [*F*_(2, 22)_ = 20.70, *p* < 0.001], indicating that reaction times differed between modality conditions. We did not find a significant interaction effect [*F*_(2, 22)_ = 0.18, *p* = 0.838]. For the purpose of *post-hoc* tests, we averaged across the levels of task for each modality condition and performed pairwise comparisons. Importantly, we found that participants were significantly faster in localizing visuotactile location cues than tactile location cues [mean difference: 0.14 s, *t*_(11)_ = −7.32, *corrected p* < 0.001], suggesting that participants did not systematically traded off speed for accuracy. On a descriptive level, participants were also faster in localizing cues in the visuotactile condition than in the visual condition (mean difference: 0.03 s) but this comparison did not reach significance [*t*_(11)_ = −1.57, *corrected p* = 0.145]. In sum, these results confirm that participants did not make a speed accuracy trade-off. On the contrary, results indicate that participants were even faster in localizing the cues when receiving visuotactile location cues in comparison to receiving only tactile location cues and on a descriptive level also faster than receiving only visual location cues.

Overall, results indicate that participants were significantly less variable in their location estimates when receiving redundant visual and tactile location in comparison to receiving only visual or tactile location cues. Moreover, the observed variance in these conditions closely matched predictions by optimal cue integration. In addition, an alternative explanation that these findings could be explained by a speed accuracy trade-off has been excluded. Crucially, these findings apply to the single as well as the dual task condition, indicating that visuotactile integration in a localization task is not affected by simultaneously performing a visual search task.

## 4. Discussion

In the present study, we investigated how multisensory processes and attentional processes are interrelated. Specifically, we investigated two research questions: (1) Are attentional resources required by a visual search task and by a tactile localization task shared or distinct? (2) Do attentional resources required by a visual search task interfere with visuotactile integration of visual and tactile location cues in a localization task? With regard to the first question, our results show that attentional resources required for a visual search task are in part distinct from the attentional resources required for a tactile localization task. With regard to the second question, our findings indicate that participants integrate location cues from the visual and tactile modality optimally regardless of additional attentional load due to a visual search task. These findings support the view that multisensory integration is an automatic process that is not dependent on attentional resources.

Possible confounds that could alternatively explain these findings have been addressed. In particular, we can exclude the possibility that participants systematically allocated their attentional resources unequally across tasks for certain task combinations that could result in systematic differences between conditions. In addition, participants did not shift their decision criteria for the search task (i.e., did not systematically searched more accurately in one condition than in another). Moreover, in the localization task, participants did not traded off speed for accuracy to localize the visuotactile location cues more accurately, thereby creating a multisensory benefit.

However, there are a few possible confounds that we did not address. In particular, our results indicate that localizing the tactile location cues was more difficult than localizing the visual location cues. Ideally, the difficulty levels of these two tasks should have been matched. During the training trials, we chose the initial parameters of the localization task such that participants subjectively felt that the tasks were equally difficult. However, this subjective impression was not reflected in the actual performance. In future studies a more careful control of performance levels is needed. However, given that the tactile localization task was more difficult than the visual localization and not vice versa, our conclusions with regard to the question whether attentional resources are distinct or shared still hold. Moreover, given our findings, we hypothesize that the search benefit for performing a visual search task and a tactile localization task in comparison to performing a visual search and a visual localization task could be even greater when the difficulty of the localization tasks would have been matched. Yet, we do want to point out that this line of argumentation is only valid under the assumption that the total amount of available attentional resources is not influenced by the task difficulty. For instance, it should not be possible to access additional attentional resources at certain difficulties of the same task that would not be accessible at other difficulty levels. We do think this assumption is reasonable as studies have shown that humans do always use their available attentional resources (for a review, see Lavie, 2005). Moreover, we assume that there is a systematic relationship between attentional demands and task performance (i.e., the more attentionally demanding the task, the lower the task performance). Given results of other studies in which a systematic relationship was found for increases in attentional demands and task performance (e.g., see Alvarez and Franconeri, 2007; Alnæs et al., 2014), we do think this assumption is reasonable as well.

Another aspect of our design that needs to be discussed are the motor components in our tasks. In particular, in the localization task, participants need to continuously press keys on the number pad to localize the given location cues while for the search task there is no ongoing demand to press keys—only when the search is aborted, participants need to press keys and at this stage they no longer need to perform the localization task. In addition to the interference in the visual search task caused by performing the localization task in different sensory modalities at the same time, it is possible that the motor actions performed in the localization task itself could interfere with the visual search task regardless of the sensory modality in which it is carried out. Therefore, the interference caused by the localization task on the visual search task may be composed of at least two components: (1) a motor component (i.e., the demand to continuously press keys) and (2) a sensory component (i.e., whether tasks are performed in the same sensory modality or different sensory modalities). We suspect that the observed interference between tasks in this study may be overestimated as it is not purely caused by the sensory component in the localization task. In a future study, in order to have a pure estimate of the interference caused by the sensory component, two tasks could be chosen that both do not require an ongoing demand to perform motor actions.

The question of whether there are distinct or shared attentional resources across sensory modalities has been addressed extensively in the literature (Duncan et al., 1997; Potter et al., 1998; Jolicoeur, 1999; Soto-Faraco and Spence, 2002; Alais et al., 2006; Hein et al., 2006; Talsma et al., 2006; Van der Burg et al., 2007; Arnell and Larson, 2002; Soto-Faraco et al., 2002; Arnell and Jenkins, 2004; Arrighi et al., 2011; Wahn and König, 2015a,b; Wahn et al., 2015). In the present study, we found that partly distinct attentional resources are used when performing a visual search task and a tactile localization task at the same time. In our earlier studies, we found a complete overlap in attentional resources for the visual and tactile modality (Wahn and König, 2015b), and also for the visual and auditory modality (Wahn and König, 2015a). However, in these studies two purely spatial tasks were performed at the same time. Yet, in other studies, it was found that there are distinct attentional resources when performing a spatial task and a discrimination task (Arrighi et al., 2011), and also distinct attentional resources when performing two discrimination tasks in separate sensory modalities (Alais et al., 2006). Taken together, a crucial aspect that determines whether shared or distinct attentional resources across sensory modalities are recruited are the type of tasks that are performed in separate sensory modalities. In particular, whether the tasks involve a discriminative component or spatial component is a determining factor. Here, our serial visual search task contains both of these components as humans need to allocate their attentional resources to different parts of the visual field (a spatial component) and once allocated, they need to discriminate targets from distractors (a discriminative component) (Eimer, 2014). In light of the mentioned previous research, the fact that we find partially overlapping attentional resources when performing a visual search task and a tactile localization task can be explained in terms of these task components. In particular, we suspect that the spatial component in the tactile localization task interfered with the spatial component in the visual search task but not with the discriminative component. Such an explanation also fits to results by a study by Alais et al. (2006) in which a visual search task was performed in combination with an auditory discrimination task. In their study, results yielded that attentional resources are distinct for the auditory and visual sensory modality. We suggest for their results that the discriminative component in the auditory discrimination task did not interfere with the discriminative component and spatial component in the visual search task as attentional resources for discrimination tasks have been found to be distinct across the sensory modalities (Duncan et al., 1997; Potter et al., 1998; Soto-Faraco and Spence, 2002; Alais et al., 2006; Hein et al., 2006; Van der Burg et al., 2007) and also distinct across sensory modalities when performing a spatial and a discrimination task at the same time (Arrighi et al., 2011).

In future studies, other types of task combinations could be tested to further elucidate how the performed type of tasks systematically influence whether shared or distinct attentional resources are used. More generally, the described dependency between the performed tasks and whether shared or distinct attentional resources are found suggests that the context (here, whether spatial or discrimination tasks are performed) in which attentional resources are studied is of vital importance. Future studies should therefore also consider other factors that could systematically affect how attentional resources are distributed across sensory modalities such as the actions that need to be performed or the type of stimuli (Engel et al., 2013).

The question whether multisensory integration is affected by attentional load has also been extensively studied in the past (Vroomen et al., 2001; Alsius et al., 2005, 2007; Zimmer and Macaluso, 2007; Helbig and Ernst, 2008; Mozolic et al., 2008; Macaluso, 2010; Gentile et al., 2013; Wahn and König, 2015a,b). While in the present study we found that optimal visuotactile integration was not dependent on attentional resources required by a visual search task, findings of previous studies have been conflicting. Many researchers indeed found that the integration process was affected by attentional load (Vroomen et al., 2001; Alsius et al., 2005, 2007; Mozolic et al., 2008); others found no effect of attentional load (Santangelo and Spence, 2007; Zimmer and Macaluso, 2007; Helbig and Ernst, 2008; Macaluso, 2010; Gentile et al., 2013; Wahn and König, 2015a,b). One reason for this discrepancy in findings could be the type of stimuli that were used in the tasks. In particular, many studies that found an effect of attentional load did use linguistic stimuli while no effect of attentional load was predominantly found when simple stimuli such as flashes or beeps were used. Yet, a different reason could be that the magnitude of attentional load manipulation was not sufficiently large in studies that did not find an effect of attentional load. However, these studies did also find considerable interference between tasks (including the present one) that makes it doubtful that the attentional load manipulation was too weak. Overall, it is not clear what factors determine how attentional load influences multisensory integration. Future studies could systematically vary the type of stimuli and/or how additional attentional load is introduced to further investigate which task factors interact with attentional load and multisensory integration. Alternatively, future studies could make use of other paradigms such as a crossmodal cueing paradigm (Spence et al., 2004; Walton and Spence, 2004) or a multisensory pattern matching task (Göschl et al., 2014, 2015) to investigate more subtle effects of attentional load. Taken together, the present findings further support the view that multisensory integration is unaffected by attentional load for non-linguistic stimuli.

Also, it should be noted that participants integrated visual and tactile location cues optimally even though the location cues on the screen and on the vibrotactile belt were considerably far apart (i.e., 90 cm). Previous research on visuotactile integration has shown that multisensory integration is negatively affected by the spatial disparity between the provided location cues (Gepshtein et al., 2005). Yet, in this study, the spatial cues from the visual and tactile modality were integrated—even when attentional resources were diverted to a simultaneously performed visual search task (also see Wahn and König, 2015b).

More generally, previous research has shown benefits for receiving tactile spatial cues especially in contexts in which little or no visual information is available (Kärcher et al., 2012), the visual sensory modality is occupied with a demanding task (Sklar and Sarter, 1999; Calhoun et al., 2002), or in contexts in which the tactile cues provide information that is normally not received such as the direction of the magnetic north (Nagel et al., 2005; Kaspar et al., 2014). The present study further promotes the use of tactile stimuli to provide directional information to recruit free attentional resources when two tasks are performed in separate sensory modalities. However, we want to highlight that the combination of the type of tasks that are performed at the same time is critical whether there are benefits for performing two tasks in separate sensory modalities.

In a recent study, we applied findings of the present study to a collaborative context (Wahn et al., 2015). In particular, co-actors in a collaborative visual search task (i.e., they were searching for a target among distractors together) mutually received the co-actor's gaze information either via the visual, auditory, or tactile modality. We found that participants performed the collaborative search task faster when receiving this gaze information via the tactile or auditory sensory modality than via the visual modality. Here, again a localization task (i.e., localizing where the co-actor is looking) is combined with a visual search task. But in this case, the location cues carry task-relevant information by another person. While this applied study also shows that doing a search task and a localization task in separate sensory modalities is beneficial compared to performing both tasks in the same sensory modality, it demonstrates that the findings of the present study generalize to a collaborative task setting. Notably, the present study also showed that multisensory integration is not dependent on attentional resources. Future studies could further apply these findings to a collaborative setting by providing task-relevant information of a co-actor via several sensory modalities, thereby making use of such robust benefits of multisensory integration.

In conclusion, the present study further supports the view that the distribution of attentional resources across sensory modalities depends on the type of stimuli that are processed and tasks that are performed. Moreover, present findings also support the view that optimal integration of spatial information from several sensory modalities is not affected by attentional load when non-linguistic stimuli are used. To further support these findings, in addition to the work that has been already done, studies could further systematically vary the type of tasks that are performed in separate sensory modalities and/or the type of stimuli that are processed.

## Author contributions

BW and PK conceived the experiment. BW conducted the experiment, analyzed the data, and wrote the manuscript. Both authors reviewed the manuscript.

## Funding

We gratefully acknowledge the support by H2020 – H2020-FETPROACT-2014 641321 – socSMCs (for BW) and ERC-2010-AdG #269716 – MULTISENSE (for PK).

### Conflict of interest statement

The authors declare that the research was conducted in the absence of any commercial or financial relationships that could be construed as a potential conflict of interest.
